# Case report of an acute myocardial infarction complicated by coronary spasm in a patient with chronic systemic lupus erythematosus

**DOI:** 10.11604/pamj.2021.38.302.27642

**Published:** 2021-03-23

**Authors:** Jeanne Lolita Pek, Zhang Jin, Sun Jian

**Affiliations:** 1 Department of Cardiology, First Hospital of Jilin University, Changchun, China

**Keywords:** Acute myocardial infarction, systemic lupus erythematosus, coronary artery spasm, case report

## Abstract

Acute myocardial infarction (AMI) is a major cause of death in a patient with systemic lupus erythematosus (SLE). Due to their chronic inflammatory state, patient with SLE has an increased risk of developing coronary artery disease. We report a case of a middle-aged woman with an acute myocardial infarction (AMI) caused by a right coronary artery (RCA) stenosis complicated with severe coronary artery spasm. Our patient has a history of long-standing SLE. Clinical expression of coronary artery disease (CAD) in SLE is the result of different pathophysiologic mechanism. From this case, we raise the importance of the clinician to be aware of the diverse pathophysiologic pathways involving a coronary artery in a patient with SLE.

## Introduction

Acute myocardial infarction (AMI) is the result of acute obstruction of the coronary artery flow leading to irreversible damage of the myocardium and therefore impairing the systolic and diastolic function of the heart. It is a major cause of death in developed countries and a great economic burden [1]. Systemic lupus erythematosus (SLE) is defined as an autoimmune disorder where the immune system strikes its own tissues leading to inflammation and damage. The chronic inflammatory state of this condition has been linked with an increase in cardiovascular disease. Systemic lupus erythematosus (SLE) can affect the coronary artery and lead to ischemic heart disease in up to 16% of the patient [2]. We report an interesting case of a middle-aged woman with a history of long-standing SLE who developed an acute myocardial infarction caused by coronary artery occlusion and vasospasm.

## Patient and observation

**Figure 1:**
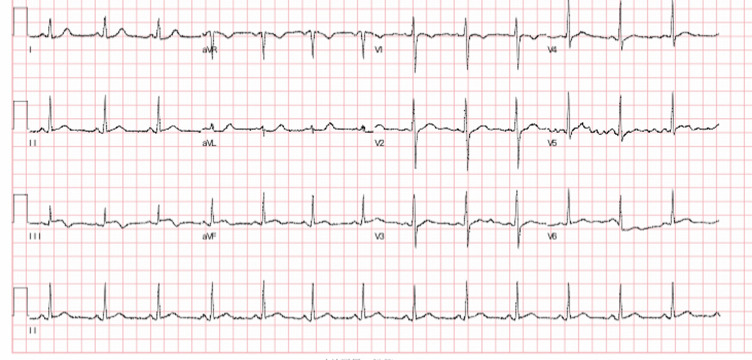
ECG showing ST-elevation in leads II, III, aVf

**Figure 2:**
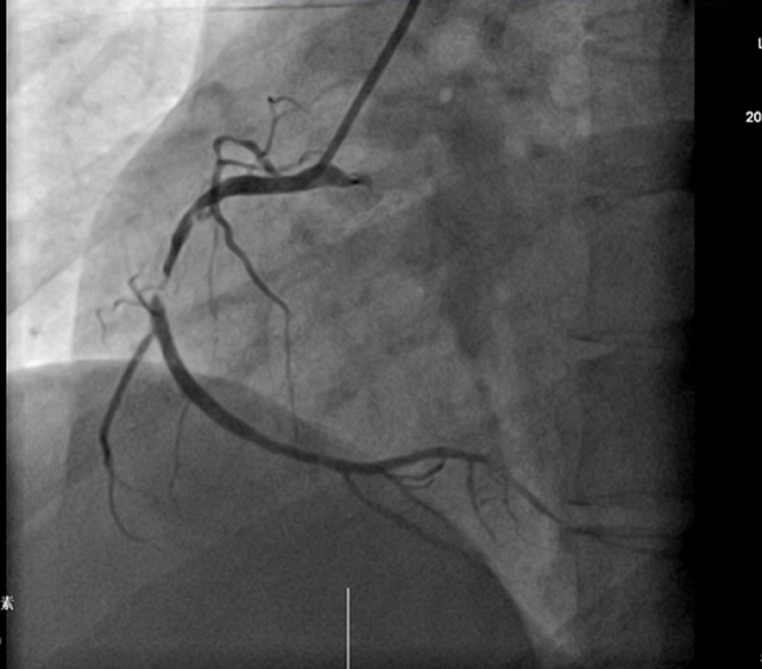
angiogram showing diffuse stenosis from proximal to middle and mid stage subtotal occlusion of the RCA

**Figure 3:**
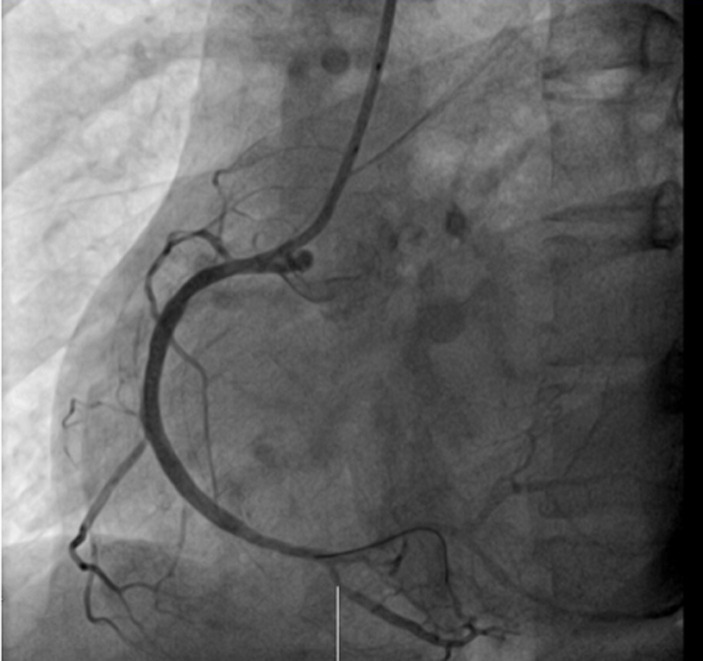
post-operative angiogram, showing a normal RCA blood flow

Thirty-year-old Asian woman was admitted to the department of cardiology with a complaint of retrosternal knife-like pain without any obvious cause. On her admission her vital signs were normal. Her past medical history is significant for hypertension, diabetes mellitus and systemic lupus erythematosus for which she has been taking prednisolone acetate tablet regularly for a year. She experienced similar pain 6 years ago and at that time the pain was relieved after administration of nitroglycerin. The electrocardiogram (ECG) on her admission (Figure 1) showed ST-segment elevation in leads II, III, and augmented vector foot (aVf) lead. On echocardiography, there was no abnormality in the structure and function of the heart, ejection fraction (EF) was 63%. The level of the cardiac troponin I was 0.22 ng/ml (>0.05), suggesting the development of an AMI. Coronary angiography was performed with the suspicion of inferior wall myocardial infarction. The angiogram showed diffuse stenosis from proximal to middle and mid stage subtotal occlusion of the RCA (Figure 2); therefore, 2 stents were placed in the RCA (Figure 3). After the surgery, the patient vitals were stable, she was then sent back to the ward where she was treated with aspirin; clopidogrel; enoxaparin; symptomatic medication and prednisolone acetate for her SLE. The day after the angioplasty with 2 stents placement, the patient complaints did not resolve, the troponin I level increased to 9.640 ng/ml and the electrocardiogram was unchanged. The rheumatology department was consulted and the patient was put on oral prednisolone acetate 27.5 mg 1/day; tacrolimus 1mg 2/day; mycophenolate 0.5 mg 2/day oral; hydroxychloroquine 0.2mg 2/day oral, based on the rheumatologist evaluation for the management of the SLE. Antinuclear antibody (ANA) panel test was performed and the anti nRNP/Sm±, ANA homogeneous pattern was positive at 1/320. The other antibodies, anti-dsDNA, Sm, SS-A and SS-B test were all negative.

**Figure 4:**
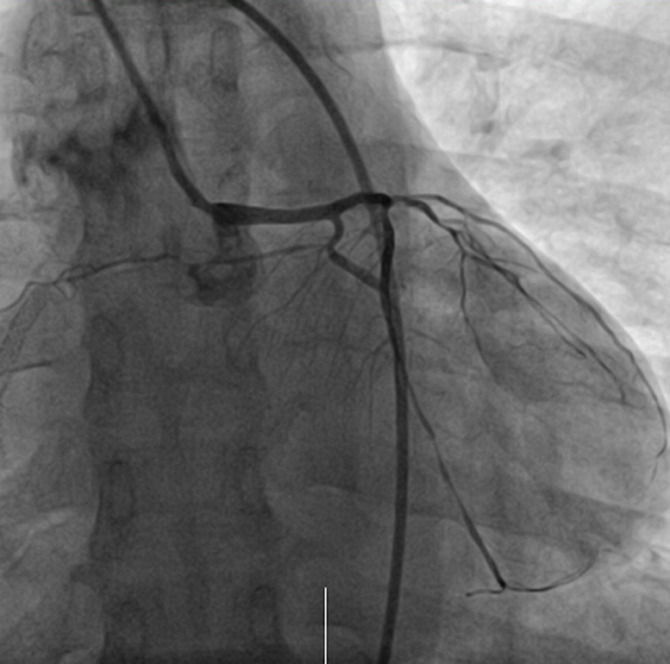
angiogram showing no thrombosis in the stent

The patient general condition further deteriorated and was then complaining of a sudden difficulty of breathing in addition to the previous substernal chest pain. Her vital signs at that time were; blood pressure, 90/65 mmHg; SO_2_, 93%; heart rate, 87 beats/min; respiratory rate; 21/min and she was transferred to the intensive care unit. Since no contraindications for surgery were found, the patient underwent a second coronary angiography to determine whether the stent was thrombosis (Figure 4). No thrombosis in the stent or any obvious narrowing was found but during the surgery, it was reported that the patient experienced coronary artery spasm that was improved after administration of nitroglycerin. This finding during the surgery and the absence of evident stenosis on the angiogram suggested that coronary artery spasm was the cause of the persistence of her symptoms. After the surgery, the patient condition ameliorated and her vitals were stable, she was then sent back to the ward. The patient was diagnosed with acute myocardial infarction exacerbate by coronary artery vasospasm. Based on a rheumatologist consult for the second time, the patient´s oral prednisolone acetate dosage was increased to 40 mg/d, the rest of the medication for SLE were unchanged. The patient was also added on a calcium channel blocker; diltiazem for the coronary spasm. Her subsequent clinical course was favorable and she was then discharged.

## Discussion

The diagnosis of AMI is usually based on the clinical presentation; the ECG findings; the laboratory results (increased in cardiac biomarker); the angiography will confirm the diagnosis [3]. Angina pectoris is the most common presentation of a patient with AMI [4] and the chest pain is typically described as a sensation of dullness, tightness or squeezing, and it most often radiates to the left arm, the lower jaw, the neck, the right arm, back and epigastrium. It can also present with other symptoms or even be asymptomatic in some cases [5]. On an ECG, ST-elevation in leads II, III, and aVf (inferior leads) usually reflect an occlusion of the right coronary artery (RCA) or in less intense an occlusion of the left circumflex (LCX). Cardiac biomarkers are enzymes that play a major role in the diagnosis of AMI. Cardiac troponin is by far the most clinically used biomarker for an early easy ruled out, risk stratification and assessment of outcome in a patient with the acute coronary syndrome. An increased of troponin I level is highly suggestive of myocardial necrosis [6]. Coronary angiography remains the most accurate diagnostic test of AMI because of its ability to visualize the coronary tree and assess its blood flow. It is also used as a treatment when stenting is necessary [7].

Our presented case provides two interesting findings. Firstly, women usually present with AMI at an advanced age (premenopausal age) and the protective mechanism is thought to be due to circulating estrogen. That mechanism is still not yet clearly understood [8]. Our patient is a middle-aged woman with a longstanding history of SLE. Due to its inflammatory effect, SLE increased the risk of developing an acute myocardial infarction [9]. Secondly, based on the clinical presentation, the laboratory and ECG findings, the observation of diffuse stenosis from the proximal to the middle, and a mid-stage subtotal occlusion in the RCA on angiography, the patient was diagnosed with an AMI caused by the RCA occlusion. Two stents were placed in. After stenting, the RCA flow was good and there was no obvious stenosis. However, recurrence of symptoms, the day after the 2 stents placement prompted for another angiogram to be performed to determine whether the stents were thrombosing, the absence of any obvious stenosis visible and the occurrence of a coronary artery spasm during the surgery enlightened us to the coexistence of another pathophysiology mechanism. Coronary spasm was most likely the cause of the patient symptoms persistence.

Systemic lupus erythematosus (SLE) can be range from a mild disease to a life-threatening condition, patients with SLE usually present with organs specific and constitutional signs and symptoms due to the systemic inflammation [10]. Chronic inflammatory state in this disorder has been linked with an acceleration of atherosclerosis, therefore with an increased incidence of cardiovascular disease [11]. Any part of the heart can be affected including the conduction system; the pericardium; the myocardium; the valves and the coronary artery. When the coronary artery is affected, the pathophysiology mechanisms leading to AMI are many and varied including atherosclerosis (most common); arteritis (second most common); thrombosis embolization; spasm. Coronary artery spasm is among the less common mechanism causing AMI in a patient with SLE and sometimes can be missed out [12].

There were two causes of the patient´s clinical presentation. The first was atherosclerosis leading to the vascular occlusion observed in the first angiogram and managed with the placement of the stents. The second was severe coronary spasm also leading to vascular occlusion, resulting in patient condition further deterioration the day after stents placement. The clinician should be aware of the possibility of coexistence of different pathophysiology mechanism in a patient with AMI. Despite the rarity, coronary artery spasm can develop in a patient with SLE and lead to acute obstruction of the coronary artery flow leading to irreversible damage of the myocardium. This mechanism is not as common as atherosclerosis and it is rarely encountered in a patient with SLE. Its actual prevalence is not well known. Better medical management of the patient SLE and administration of calcium channel blocker (vasodilator) for the spasm improve the patient condition [13].

## Conclusion

The clinical physician should be aware of the increase in the incidence of a cardiovascular event in a patient with chronic SLE. Therefore, preventives measure for coronary heart disease should be mandated in a patient with SLE. There is a different mechanism in which AMI may develop. Coronary artery spasm although less common than atherosclerosis should also be ruled out in a patient with a history of SLE. Those patient´s coronary spasm can be well medically managed with vasodilator.

## References

[1] Mechanic OJ, Grossman SA (2020). Acute myocardial infarction.

[2] Farooq A, Ullah A, Ali F, Yasin H, Amjad W, Pervaiz M (2017). Acute myocardial Infarction in young systemic lupus erythematosus patient with normal coronary arteries. Cureus.

[3] Pandey R, Gupta NK, Wander GS (2011). Diagnosis of acute myocardial infarction. J Assoc Physicians India.

[4] Wilmot KA, O´Flaherty M, Capewell S, Ford ES, Vaccarino V (2015). Coronary heart disease mortality declines in the United States from 1979 through 2011: evidence for stagnation in young adults, especially women. Circulation.

[5] Aghdam MRF, Vodovnik A, Sund BS (2016). Sudden death associated with silent myocardial infarction in a 35-year-old man: a case report. Journal of medical case reports.

[6] Apple FS, Sandoval Y, Jaffe AS, Ordonez-Llanos J, IFCC Task Force on Clinical Applications of Cardiac Bio-Markers (2017). Cardiac troponin assays: guide to understanding analytical characteristics and their impact on clinical care. Clin Chem.

[7] Williams MC, Hunter A, Shah AS, Assi V, Lewis S, Smith J (2016). Use of coronary computed tomographic angiography to guide management of patients with coronary disease. Journal of the American College of Cardiology.

[8] Matthews KA, Santoro N, Lasley B, Chang Y, Crawford S, Pasternak RC Relation of cardiovascular risk factors in women approaching menopause to menstrual cycle characteristics and reproductive hormones in the follicular and luteal phases. The Journal of Clinical Endocrinology and Metabolism. 2006;.

[9] Iaccarino L, Bettio S, Zen M, Nalotto L, Gatto M, Ramonda R (2013). Premature coronary heart disease in SLE: can we prevent progression. Lupus.

[10] Cojocaru M, Cojocaru IM, Silosi I, Vrabie CD (2011). Manifestations of systemic lupus erythematosus. Maedica (Bucur).

[11] Esdaile JM, Abrahamowicz M, Grodzicky T, Li Y, Panaritis C, du Berger R (2001). Traditional Framingham risk factors fail to fully account for accelerated atherosclerosis in systemic lupus erythematosus. Arthritis Rheum.

[12] Doria A, Iaccarino L, Sarzi-Puttini P, Atzeni F, Turriel M, Petri M (2005). Cardiac involvement in systemic lupus erythematosus. Lupus.

[13] Moder KG, Miller TD, Tazelaar HD (1999). Cardiac involvement in systemic lupus erythematosus. Mayo Clinic Proceedings.

